# Exosomes derived from microRNA-129-5p-modified tumor cells selectively enhanced suppressive effect in malignant behaviors of homologous colon cancer cells

**DOI:** 10.1080/21655979.2021.2004981

**Published:** 2021-12-09

**Authors:** Yong Ji, Jianxiang Ji, Hongming Yin, Xu Chen, Pengcheng Zhao, Huahu Lu, Taowu Wang

**Affiliations:** Department of Surgery, Funing County Hospital, Yancheng, Jiangsu, China

**Keywords:** Homologous colon cancer, exosomes, miRNA-129-5p, gene expression, molecular mechanism

## Abstract

Exosome-encapsulated microRNAs (miRNAs) are novel diagnostic and predictive markers in colon cancer. Hence, the study of serum exosomal miRNAs in patients with colon cancer may help its diagnosis and treatment. PKH26-labeled exosomal uptake analysis identified whether exosomes transfer miRNA-129-5p to target cells. Transmission electron microscopy and dynamic light scattering analysis were applied to determine exosome morphology and size distribution. The Cell Counting Kit-8, wound healing assay and Transwell assays were used to detect cell proliferation, migration, and invasion after treatment with engineered exosomes. Moreover, the Western blotting was used to quantify the expression of proteins involved in cell apoptosis. In our study, hepatocellular liver carcinoma, cervical cancer and colon cancer cells were selected as the target cells of miRNA-129-5p exosomes. Exosomes containing miRNA-129-5p were found to be significantly more easily absorbed by colon cancer cells, presenting a stronger inhibitory effect on colon cancer cell proliferation. MiRNA-129-5p exosomes induced apoptosis in colon cancer cells while inhibiting their proliferation, migration, and invasion. In conclusion, exosomes derived from miRNA-129-5p-modified tumor cells selectively inhibited colon cancer progression, shedding new insights to therapeutic efficacy of this cancer.

## Introduction

Colon cancer is one of the most common cancers worldwide, and its prevalence has been increasing in recent years [1,2]. Due to its aggressiveness, poor prognosis, and lack of targeted treatment, colon cancer shows a high incidence rate [3]. Recently, many studies have reported that changes in miRNA expression or miRNA dysfunction may influence the occurrence and development of tumors, as well as clarified the molecular mechanism of miRNAs in tumors [4].

MicroRNAs (miRNAs) are single-stranded RNA sequences that regulate gene expression at the post-transcriptional level and play essential roles in the onset and progression of many diseases [5]. Increasing evidence clearly shows that miRNAs are related to a variety of cancers [6–10]. For instance, Qiu *et al*. discovered that miRNA-129-5p inhibits pancreatic cancer cell proliferation, migration, and apoptosis by targeting pre-leukemia transcription factor 3 [11]. Wu *et al*. discovered that metastasis-associated lung adenocarcinoma transcript 1 regulates high mobility group box-1 *in vitro* by sponging miRNA-129-5p, influencing the development of colon cancer [12]. MiRNA-129-5p is released from exosomes and delivered to target cells, regulating cell differentiation, proliferation, and apoptosis [13–15]. However, the current knowledge of exosomal miRNA-129-5p in colon cancer is limited.

Exosomes are vesicle-like particles secreted by cells with a diameter of 40–100 nm [16]. Exosomes from different cells have been detected to contain numerous mRNAs, miRNAs and proteins [17,18]. Since exosomes are naturally derived from cells and can be absorbed, they are easy to obtain tumor targeting ability through molecular biology methods [19]. Inspired by this, an increasing number of scholars have focused their research on natural exosomes to deliver drugs or functional nucleic acids to specific disease-related tissues in order to achieve better curative effects [20,21]. Many studies indicated that exosomes promote tumor metastasis as well [22–24].

In the study, we found that exosomes were easy to be taken up by colon cancer cells. A hypothesis was made that exosome-delivered miRNA-129-5p plays a significant role in colon cancer. The effects of miRNA-129-5p derived from exosomes on the proliferation, apoptosis, migration, and invasion of colon cancer cells were assessed. Our findings indicated that miRNA-129-5p-modified tumor cell-derived exosomes exert an inhibitory effect on malignant behaviors of colon cancer cells.

## Materials and methods

### Cell culture

The American Type Culture Collection provided human hepatocellular liver carcinoma cell line HepG2, cervical cancer cell line HeLa and colon cancer cell line HT29, which were cultured in DMEM supplemented with 10% fetal bovine serum (FBS; Gibco; Thermo Fisher Scientific, Inc.), 2 mmol/L glutamine (Gibco; Thermo Fisher Scientific, Inc.), 100 U/mL penicillin and 100 mg/mL streptomycin (Gibco; Thermo Fisher Scientific, Inc.) at 37°C with humidified air with 5% CO_2_.

### Exosome isolation and quantification

Exosomes released by HepG2, Hela, and HT29 cells after 24 h of culture were extracted from culture medium supplemented with 10% FBS by different centrifugations. Isolated vesicles were purified on a 30% sucrose/D_2_O cushion. The sucrose cushion vesicles were recovered, washed, and ultracentrifuged for 90 min in PBS before being collected for use. The protein content of exosomes was determined using the Bradford method. All experiments were performed in accordance with the manufacturer’s instructions.

### Exosome uptake analysis with PKH26 labeling

Exosomes were labeled using a PKH26 Red Fluorescent Cell Linker Kit (Sigma-Aldrich) as instructed by the manufacturer. The cell nuclei were stained with a solution of 6-diamidino-2-phenylindole (DAPI). The sample in the slide was examined using a laser scanning microscope (Carl Zeiss Meditec, Jena, Germany). A fluorescence microscope (DMI4000B; Leica) was used to detect the uptake of labeled exosomes by recipient colon cancer cells.

### Transmission electron microscopy (TEM)

TEM was used to examine the morphology of exosomes. The protocol is: 1) exosomes suspension was fixed in 4% paraformaldehyde. 2) this mix was transferred to the TEM grid (HT7700, Hitachi, Tokyo, Japan). The exosome samples were examined using a Hitachi H-7650 transmission electron microscope (Tokyo, Japan).

### Dynamic light scattering (DLS) analysis

DLS (Zetasizer Nano ZS90) experiment was used to determine exosome size distribution. The size distribution, that is, the hydrodynamic diameter (Dh) distribution, is obtained using the Stokes-Einstein relation: d = (kBT)/(3πηDh), where d is the diffusion coefficient, kB is the Boltzmann constant, η is the medium viscosity and T is the absolute temperature. The average hydrodynamic diameter of the exocrine body is calculated by fitting the Gaussian function to the measured size distribution. Distribution P (D) of the diffusion coefficient D can be determined using the constrained regularization method or the gamma distribution the stokes-Einstein relation is used to calculate the size distribution, that is, the hydrodynamic diameter Dh distribution: d = (kBT)/(3Dh), where d is the diffusion coefficient, kB is the Boltzmann constant, and T is the temperature. Fitting the Gaussian function to the measured size distribution yields the average hydrodynamic diameter of the exosome.

### Reverse transcription quantitative polymerase chain reaction (RT-qPCR)

Total RNA was isolated from cells using the TRIzol reagent (Invitrogen) and reverse transcriptase was used to convert it to complementary DNA. SYBR Green PCR Master Mix was used for RT-qPCR (Takara, Kyoto, Japan). The relative expression of miRNA-129-5p was calculated by the 2^−ΔΔCT^ method [25] and U6 was used as an internal reference. The primer sequences for RT-qPCR were listed as follows: miRNA-129-5p: 5ʹ-CTTTTTGCGGTCTGGGCTTG-3ʹ (forward), 5ʹ-AACGCTTCACGAATTTGCGT-3ʹ (reverse); U6: 5ʹ-CTCGCTTCGGCAGCACA-3ʹ (forward), 5ʹ-AACGCTTCACGAATTTGC-3ʹ (reverse).

### Cell counting kit- 8 (CCK-8) assay

The CCK-8 assays were performed to detect cell viability using a CCK-8 kit (Dojindo, Shanghai, China) according to the manufacture’s protocol. Briefly, cells (5 × 10^3^ cells/well) were seeded into 96-well plates and cultured at 37°C with 5% CO_2_ for 24, 48, and 72 h. Then, at different points, 10 μL of CCK8 reagent was added to each well, mixed uniformly and cultured for another 2 h avoiding lights at room temperature. The optical absorbance at 450 nm was measured using a microplate reader (Thermo Fisher Scientific Inc.).

### Transwell assays

The migration and invasion capabilities of cells were investigated by Transwell assays. Briefly, normal Transwell chamber and Matrigel precoated Transwell chamber were adopted for migration and invasion assays, respectively. Transfected cells in serum-free medium were seeded into the upper chamber, and culture medium containing 10% FBS was coated on the lower chamber. After incubating at 37°C for 24 h, the Transwell was removed, and cells were stained with 0.1% crystal violet (Sigma; Merck KGaA) at 37°C for 5 min. The number of migrated and invaded cells in the lower chamber was counted using a fluorescence microscope (magnification, 40).

### Scratch wound-healing assay

To assess the ability of cells to migrate laterally, scratch wound-healing assays were performed using the previously described method [26]. Briefly, cells were plated in 6-well plates until cells reached 100% confluency. After washing with PBS, the serum-free medium was added to each well. Wounds were scratched using a 20 μL sterile pipette tip. Then, the scratched cells were cultured for 48 h, and independent images were taken of each well at 0 h and 48 h. The wound width was observed.

### Western blot analysis

Western bolt analysis was applied to detect the protein expression levels of apoptosis indexes and exosomal markers. The BCA protein assay was adopted as a standard to determine the protein content. Protein samples were separated using sodium dodecyl sulfate-polyacrylamide gel electrophoresis and transferred to nitrocellulose membranes. The membrane was blocked with 50% fat-free milk and incubated overnight with the appropriate primary antibodies including anti-Bcl-2 (sc-7382, 1:1000), anti-Bax (sc-7480, 1:1000), anti-β-actin (sc-8432, 1:5000) (SantaCruz Biotechnology, CA, USA), anti-cleaved caspase-3 (9668, 1:1000) and anti-cleaved caspase-9 (9509, 1:1000) (Cell Signaling Technology, Beverly, MA, USA). After fully rinsing with Tris Buffered Saline Tween containing 0.1% Triton×100 buffer solution, the membrane was incubated with secondary antibody (sc-69,786, 1:1000; Santa Cruz Inc, Santa Cruz) at room temperature for 1 h. Then, the protein bands were visualized by an Ultra High Sensitivity ECL Substrate Kit (ab133409, Abcam, Shanghai, China). The semi-quantitative results were obtained by quantification of optical density using the ImageJ software.

### Statistical analysis

Each experiment was conducted in triplicates. The data was analyzed using GraphPad Prism (Graph-Pad Prism, San Diego, CA, USA). All data were expressed as the mean ± standard deviation. Student’s t-test was used to compare data between two groups, and one-way analysis of variance was used to compare data from multiple groups. *P* < 0.05 was considered statistically significant.

## Results

### Characterization of exosomes

**Figure 1.**
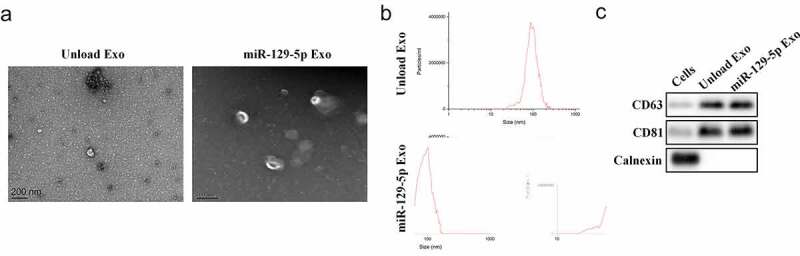
**Preparation and identification of engineered exosomes**. (a) Exosome characterization was observed using TEM. (b) DLS experiments assessed the size distribution of exosomes. (c) The protein expression of CD63, CD81, and Calnexin

We first collected and isolated exosomes from tumor cells and verified the exosomes. Exosomes are small membrane-bound vesicles with diameters ranging from 60 to 150 nm that can be seen under an electron microscope (Figure 1a). DLS analysis indicated that the average exosome size was 100 nm (Figure 1b). Western blot analysis provided evidence suggesting that the particles expressed exosome surface markers including CD63 and CD81, but not Calnexin (Figure 1c), indicating that these particles were indeed exosomes.

### Cellular uptake of autologous-Exosomes

**Figure 2.**
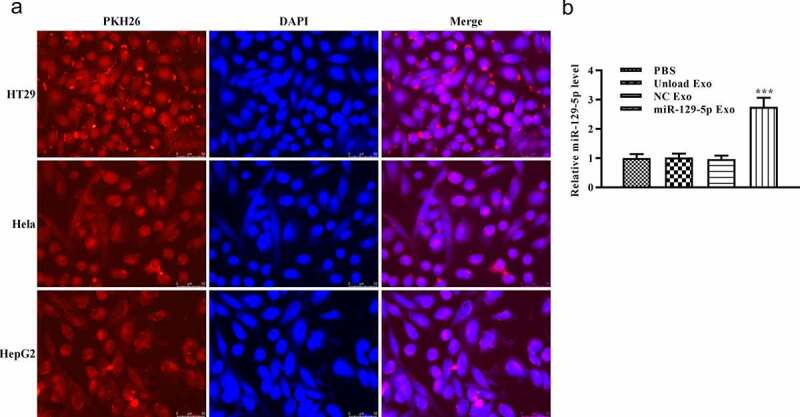
**Specific uptake of autogenous exocrine by tumor cells**. (a) Colon cancer cells were co-cultured with cancer cell-derived exosomes for 24 h. The exosome uptake of colon cancer cells was confirmed by a confocal microscope (scale, 10 μm). Exosomes were stained by PKH26 (red). Cell nuclei were stained by DAPI (blue). (b) The levels of miR-129-5p were detected by RT-qPCR analysis. ***P < 0.001 vs NC Exo

Subsequently, we used PKH26 to mark the exosomes and examined the existence of PKH26-labeled exosomes in cells. PKH26 dye (red) and DAPI dye (blue) were used to label the exosome and cell nucleus, respectively. After 24 h of culture, PKH26-labeled exosome was effectively internalized by adherent colon cancer cell lines, according to microscopic analysis (Figure 2a). In addition, we detected the relative expression of miRNA-129-5p by RT-qPCR. According to the result, the relative expression of miRNA-129-5p in the miRNA-129-5p Exo group was significantly increased when compared with PBS, unload Exo, and NC Exo groups (Figure 2b, *p* < 0.005). These results indicated that exosomes could be taken up by colon cancer cells and was majorly located in the cytoplasm.

### MiRNA-129-5p exosomes inhibit tumor cell proliferation

**Figure 3.**
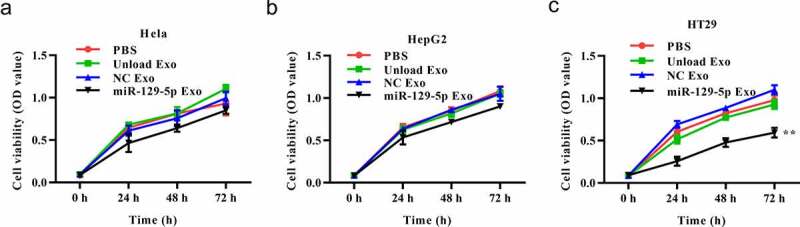
**Effects of miR-129-5p-loaded exosomes on tumor cell viability**. (a-c) The cell viability was assessed by CCK8 assay in Hela, HepG2, and HT29 cells. **P < 0.01 vs NC Exo

To better understand the effect of the miRNA-129-5p-loaded exosomes in tumor cells, we conducted a series of *in vitro* functional experiments. In the CCK-8 assays, the cells (HepG2, Hela, and HT29) were treated with PBS, unloaded exosome, NC mimic-loaded exosome or miRNA-129-5p-loaded exosome. The results exhibited that unloaded or NC mimic-loaded exosomes had no significant effects on the viability of Hela, HepG2 and HT29 cells. MiRNA-129-5p-loaded exosome caused no significant effects on the viability of Hela, HepG2 cells, but exerted an inhibitory effect on HT29 cell viability (Figures 3A-3 C).

### Effect of miRNA-129-5p exosomes on apoptosis-related proteins in colon cancer cells

**Figure 4.**
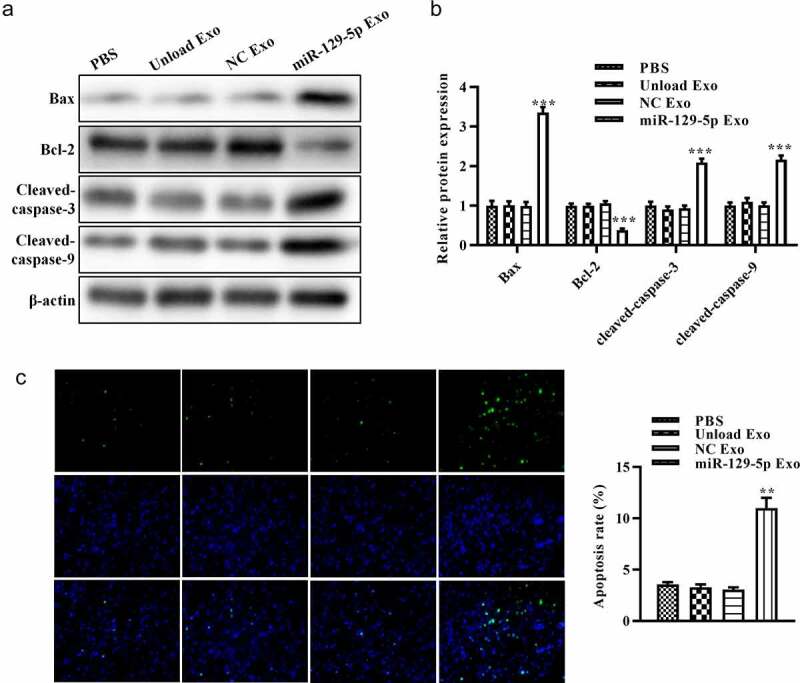
**Effect of miR-129-5p-loaded exosomes on apoptosis of tumor cells**. (a) The protein levels of Bcl-2, Bax, cleaved caspase-3, and cleaved caspase-9 in colon cancer cells were revealed by Western blot analysis. (b) The relative protein expression levels were quantified by the ImageJ software. (c) TUNEL staining assay was performed on colon cancer cells after treatment with PBS, unloaded exosomes, NC mimics-loaded exosomes, and miR-129-5p-loaded exosomes. **P < 0.01, ***P < 0.001 vs NC Exo

To further assess the effect of miRNA-129-5p-loaded exosomes on apoptosis of HT29 cells, we evaluated the expression of apoptosis-related proteins (Bcl-2, Bax, cleaved-caspase-3, and cleaved-caspase-9). The results of Western blot analysis showed that, compared with unloaded or NC mimic-loaded exosomes, miRNA-129-5p-loaded exosomes reduced Bcl-2 expression and increased the expression of Bax in tumor cells. Furthermore, miRNA-129-5p-loaded exosomes induced the activation of cleaved-caspase-3 and cleaved-caspase-9 (Figures 4A and 4B). Figure 4c revealed that miRNA-129-5p-loaded exosomes significantly suppressed the apoptosis rate of HT29 cells. These results indicated that exosomes derived from miRNA-129-5p-modified tumor cells may play an important role in inducing HT29 cell apoptosis.

### Effect of miRNA-129-5p exosomes on migration and invasion of tumor cells

**Figure 5.**
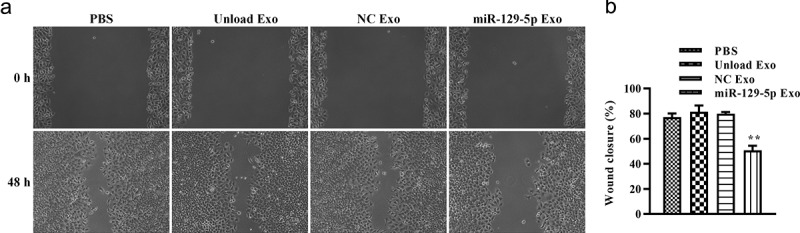
**Effect of miR-129-5p-loaded exosomes on tumor cell migration**. (a) The lateral migration of colon cancer cells was analyzed using a scratch wound-healing assay. (b) The percentage of wound closure of colon cancer cells. **P < 0.01 vs NC Exo

**Figure 6.**
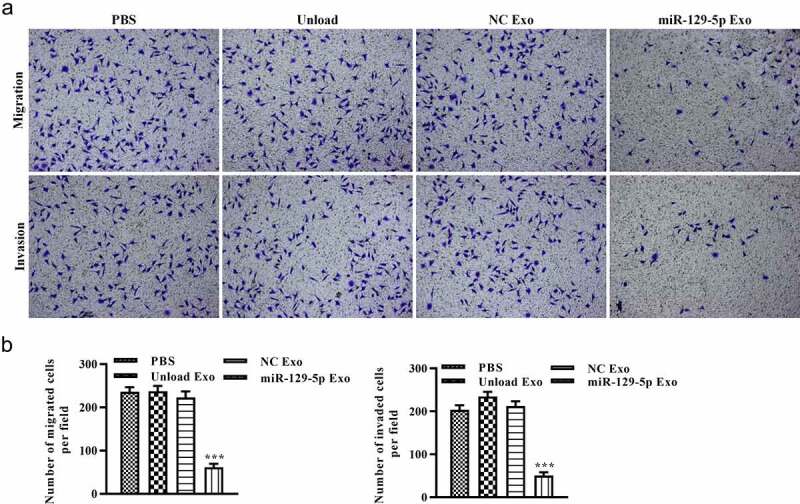
**Effect of miR-129-5p-loaded exosomes on tumor cell migration and invasion**. (a) The migration and invasion of colon cancer cells was analyzed by Transwell assays. (b) The number of migrated and invaded cells. ***P < 0.001 vs NC Exo

To explore the potential of miRNA-129-5p exosomes in tumor cell migration and invasion, scratch wound healing and Transwell assays *in vitro* were performed. As shown in Figures 5A and 5B, after 48 h, the distance of tumor cell scratches was significantly wider in miR-129-5p Exo group than the PBS, unload Exo, and NC Exo groups. Interestingly, the Transwell migration assays revealed that the cells in the miRNA-129-5p Exo group had a lower degree of motility compared with those in PBS, Unload Exo, and NC Exo groups (Figure 6a). In addition, the results of the Transwell invasion assays indicated that the invasion ability of cells in the miRNA-129-5p Exo group was evidently lower than cells in the PBS, unload Exo and NC Exo groups (Figure 6a). Statistical analysis showed that the number of migrated and invaded cells per field was significantly reduced in the miRNA-129-5p Exo group compared with the other groups (Figure 6b).

## Discussion

Colon cancer, a most common gastrointestinal cancer, is caused by uncontrolled cell growth in the colon or rectum [27]. As patients with advanced cancer are prone to abnormal cell proliferation and migration, inhibiting the growth and migration of colon cancer cells can prolong the survival of colon cancer patients [28]. MiRNA-129 family is composed of miRNA-129-5p (previously miRNA-129), miRNA-129-1-3p (previously miRNA-129*) and miRNA-129-2-3p (previously miRNA-129-3p) [29]. The current papers on miRNA-129-5p mainly focused on tumors, for instance, research by Ge *et al*. showed that miRNA-129-5p functions as a tumor suppressor in non-small cell lung cancer through high mobility group box-1 [30]. Moreover, miRNA-129-5p has been demonstrated to stimulate cell proliferation and invasion in breast cancer [31]. In the present study, we found that miRNA-129-5p-loaded exosomes showed the inhibitory effects in colon cancer cells.

To investigate the ability of exosomes to target homologous tumor cells, we designed cells expressing fluorescence-labeled exosomes and incubated homologous tumor cells with engineered exosomes containing miRNA-129-5p to ensure that the exosomes could transfer miRNA-129-5p to target cells. Our findings showed that exosomes successfully transferred miR-129-5p to target cells, and the expression was the highest in the miRNA-129-5p Exo group. Previous studies have shown that miRNA-129-5p is down-regulated in colon cancer tissues, and miRNA-129-5p mimics can inhibit the growth of colon cancer cells [12].

The relationship between the expression of miRNA-129-5p and the progression of colon cancer is still considered to be a complicated content. Moreover, the current research on the function of miRNA-129-5p as exosomes on colon cancer is rarely involved. Accordingly, we clarified the function of miRNA-129-5p exosomes on colon cancer cells through a series of *in vitro* experiments. In this paper, we used CCK-8 assay to examine the effect of miRNA-129-5p-loaded exosomes on viability of cancer cells, and the results clearly showed that miRNA-129-5p-loaded exosomes strongly inhibited the growth of colon cancer cells, but did not cause significant inhibitory effects on hepatocellular liver carcinoma and cervical cancer cells. Further experimental results depicted that miRNA-129-5p-loaded exosomes inhibited the migration, invasion and promoted apoptosis of colon cancer cells. Thus, miRNA-129-5p in exosomes might serve as a potential novel biomarker for colon cancer diagnosis. However, the levels of serum exosomal miRNAs in colon cancer do not necessarily represent their cellular levels, since the miRNA sorting mechanism may affect the incorporation of miRNAs into exosomes [32]. At the disease level, the increase in circulating miRNA levels may not be the result of the upregulation of miRNAs in the pathological tissues, but be the negative impact of the disease on the expression of miRNAs in other cells.

## Conclusion

Exosomal miRNA-129-5p inhibited the malignant behaviors of colon cancer cells, which provides a new detection of serum biomarkers in the pathogenesis and diagnosis of colon cancer.

## Data Availability

The datasets during and/or analyzed during the current study available from the corresponding author on reasonable request.
